# Medical Lexicon of Modern Terminology

**Published:** 1855-10

**Authors:** 


Art. VI.—*Medical Lexicon of Modern Terminology*; being a complete Vocabulary of Definitions, including all the Technical Terms employed by Writers and Teachers of Medical Science at the present day, and comprising several hundreds of words not found in any other Dictionary. Third Edition. By D. Meredith Reese, M. D., LL. D., Resident Physician of Bellevue Hospital, N. Y., etc., etc. New York: Samuel S. & Wm. Wood, 261 Pearl street. 1855. 12mo., pp. 233.

---

As stated in the preface, this little Lexicon "is simply a vocabulary of definitions," compressed in the smallest possible space and made of the briefest possible character consistent with perspicuity. It contains no reference to the etymology of terms and omits many obsolete technicalities and ancient terminological nomenclatures. But it presents, in the highest state of condensation nearly every term retained by modern authors or employed by most medical teachers. It is small; it is neat; it is handy; it is well printed and neatly bound. D. W. Y.

